# Physiological and oxidative stress response of carrot (*Daucus carota* L.) to jumping plant-louse *Bactericera trigonica* Hodkinson (Hemiptera: Psylloidea) infestation

**DOI:** 10.1186/s12870-024-04946-4

**Published:** 2024-04-04

**Authors:** Marija Đurić, Slađana Jevremović, Milana Trifunović-Momčilov, Snežana Milošević, Angelina Subotić, Dušanka Jerinić-Prodanović

**Affiliations:** 1 Department for Plant Physiology at the Institute for Biological Research “Siniša Stanković”, - National Institute of Republic of Serbia, University of Belgrade, Bulevar Despota Stefana 142, Belgrade, 11108 Serbia; 2 Department of Entomology and Agricultural Zoology, Faculty of Agriculture, University of Belgrade, Nemanjina 6, Belgrade, 11080 Serbia

**Keywords:** *Daucus carota*, Biotic stress, *Bactericera trigonica*, Antioxidant defense system

## Abstract

**Background:**

Carrot is an important vegetable crop grown worldwide. The major economic problem in carrot cultivation is yellow disease caused by *Bactericera trigonica*, which induces biotic stress and has the greatest impact on crop productivity. Comprehensive studies on the mechanism of carrot defense response to biotic stress caused by *B. trigonica* infestation have yet to be conducted.

**Methods:**

The changes in photosynthetic pigments, proline, TPC, H_2_O_2_ and MDA content, DPPH radical scavenging ability, and antioxidant enzyme activity of SOD, CAT, and POX in carrot leaves in response to insect sex (female and male), rapid response (during the first six hours), and long-term response to *B. trigonica* infestation were evaluated.

**Results:**

The results of our study strongly suggest that *B. trigonica* infestation causes significant changes in primary and secondary metabolism and oxidative status of carrot leaves. Photosynthetic pigment content, TPC, and DPPH and CAT activities were significantly reduced in carrot leaves in response to insect infestation. On the other hand, proline, H_2_O_2_ content, and the activity of the antioxidant enzymes superoxide dismutase and peroxidase were increased in carrot leaves after *B. trigonica* infestation. The results indicate that *B. trigonica* attenuates and delays the oxidative stress responses of carrot, allowing long-term feeding without visible changes in the plant. Carrot responded to long-term *B. trigonica* infestation with an increase in SOD and POX activity, suggesting that these enzymes may play a key role in plant defense mechanisms.

**Conclusions:**

This is the first comprehensive study strongly suggesting that *B. trigonica* infestation causes significant changes in primary and secondary metabolism and an attenuated ROS defense response in carrot leaves that enables long-term insect feeding. The information provides new insights into the mechanisms of carrot protection against *B. trigonica* infestation.

## Introduction

Carrot (*Daucus carota* L.) is a biennial herbaceous species belonging to the Apiaceae family which originating in Central Asia [1]. Because of the tasty and nutritious taproot carrot is cultivated worldwide and represent one of the most important and popular vegetable crops [2]. The storage root of carrot is rich source of valuable nutrients such as carotenoids, dietary fibers, vitamins and antioxidants [3]. The consumption and production of carrots are increasing in recent years as this vegetable is recognized as a valuable source of natural antioxidants (β-carotene) with anticancer, free radical scavenging, antimutagenic, and immune-boosting effects [2]. The major economic problem in carrot cultivation is yellow disease with symptoms such as curling and discoloration of leaves (yellowish, bronze, purple leaves), stunting of shoots and roots, proliferation of secondary roots, and altered (bitter) taste [4]. Leaf yellowing is the main symptom very often associated in the past with the transmission of the fastidious alpha-proteobacterium "*Candidatus* Liberibacter solanacearum". This bacterium can be transmitted in carrot by the insect vectors *Bactericera trigonica*, Hodkinson 1981 [5–7] and *Trioza apicalis* Föster [8, 9]. Because of the similarities between symptoms caused by insect feeding and symptoms of pathogens, there is considerable overlap between plant responses to pathogens and plant responses to insects [10]. However, recent studies have clearly shown that the observed symptoms on carrot leaves and the change in root quality are mainly caused by *B. trigonica* infestation and not by the presence of proteobacterium [4].

*B. trigonica*, the jumping plant louse, is a psyllid belonging to the order Hemiptera (Psylloidea, Triozideae), which includes about 4,000 described species [11]. Some Hemiptera species are considered as serious pests in agriculture and forestry [12–14]. *B. trigonica* was described over forty years ago based on analysis of imago collected from carrots grown in Portugal, Italy, Cyprus, Turkey, Egypt, and Iran [15]. Later, the insect was also recorded in other parts of Europe [16], in Israel [17], and in all carrot-growing areas in Serbia [18, 19]. If psyllids are not controlled, overwintering females can cause 100% yield loss in carrots [20, 21]. *B. trigonica* belongs to a group of sap-sucking insects or "phloem feeders" that take up nutrients from the phloem to complete their life cycle on a particular host plant [14, 22]. Phloem feeders can affect host growth and development by feeding and excreting toxins with saliva [23, 24], laying eggs on them, or serving as bacterial or viral vectors for plants [6, 25]. When various plant parts are mechanically damaged, phloem feeders impair critical functions such as water and mineral uptake by roots, photosynthesis and transpiration, pigment content, sugar metabolism, oxidative status, and reproduction in many plant species [26–32]. The life cycle of *B. trigonica* is last about one month and several generations during the year can be produced in the field. Imago and nymphs of *B. trigonica* feed on carrot leaves cause chlorosis and leaf yellowing, and when present in larger numbers, lead to lower crop productivity, yield, and root quality [4, 19]. Under laboratory conditions, they can have up to 9 generations during a year [19]. Recent studies have shown that plant age, temperature, and insect sex have a crucial influence on the damage intensity caused by *B. trigonica* infestation on carrot [4, 7, 33].

In addition to abiotic environmental stress, insect feeding causes biotic stress in the host plant, which has the greatest impact on plant productivity. Due to the ongoing competition between plants and insects for more than 350 million years, both have evolved defense mechanisms to circumvent each others defense systems [34]. Plants employ various morphological, biochemical, and molecular strategies to respond to insects and mitigate their harmful effects. These mechanisms include structural features such as spines, trichomes, thick epidermal layers, specialized secondary metabolites that can disrupt insect attacks in various ways, and attraction of natural enemies of the target insects [35, 36]. Numerous physiological and biochemical reactions may be involved in plant defense mechanisms against insects. Secondary metabolites play one of the most important roles in plant–insect interactions, and are produced both constitutively and as an induced response of plants to insect feeding, reviewed in Fürstenberg-Hägg et al. [37]. In addition, reactive oxygen species (ROS) content may be increased in plant tissues after insect attack. Changed ROS production after insect attack may lead to alterations in the antioxidant defense system in some plants. On the other hand, the increased ROS production could help plants to overcome the insect attack, but it could also affect the plants own physiological state and cause damage to proteins, lipids, and nucleic acids [38]. In addition to enzymatic antioxidant reactions, plants possess a variety of non-enzymatic compounds that play important roles in counteracting oxidative stress, such as carotenoids, proline, and phenolic compounds [38–41]. Peroxidation of membrane lipids is one of the well-studied parameters in response to increased ROS due to insect attack and is usually manifested by an increased product of lipid peroxidation—malondialdehyde (MDA) [42]. Accordingly, there is a need for ROS removal by the components of the antioxidant defense system and a balance between produced and removed ROS forms. In recent years, oxidative changes and the activation of antioxidant enzymes in plants after insect attack have received a lot of attention. The increase or decrease of various physiological and oxidative stress responses to feeding by different insects can vary depending on the type of insect feeding (chewing or phloem –feeding) and has been studied in many plant species [43–47]. There is a lack of data considering the physiological and oxidative stress responses of carrot to *B. trigonica* infestation. Previous studies on carrot response to *B. trigonica* infestation focused mainly on pest control and biology [4, 7]. The newest data, where symptomatic carrot plants were grouped by degree of damage in the field, indicate the importance of nymph density on flavonoid, total polyphenol and photosynthetic pigment content in carrot plants in response to *B. trigonica* infestation [4].

The main objective of this study is a comprehensive investigation of carrot defense mechanism in response to biotic stress caused by *B. trigonica* infestation. A comparative analyses of the physiological and oxidative stress responses of carrot to insect individuals of different genders and to short- (during first six hours) and long-term (after four and 28 days) *B. trigonica* infestations were performed using individuals of both genders. The oxidative response was assessed by measurements of oxidative stress by-products and enzymatic and non-enzymatic components of the antioxidant defense system, with the hypothesis of how and at what level these parameters may be altered in response to *B. trigonica* infestation.

## Results

### Physiological and oxidative stress response of carrot to *B*. *trigonica* infestation: the influence of insect gender

#### Photosynthetic pigments content

**Fig. 1:**
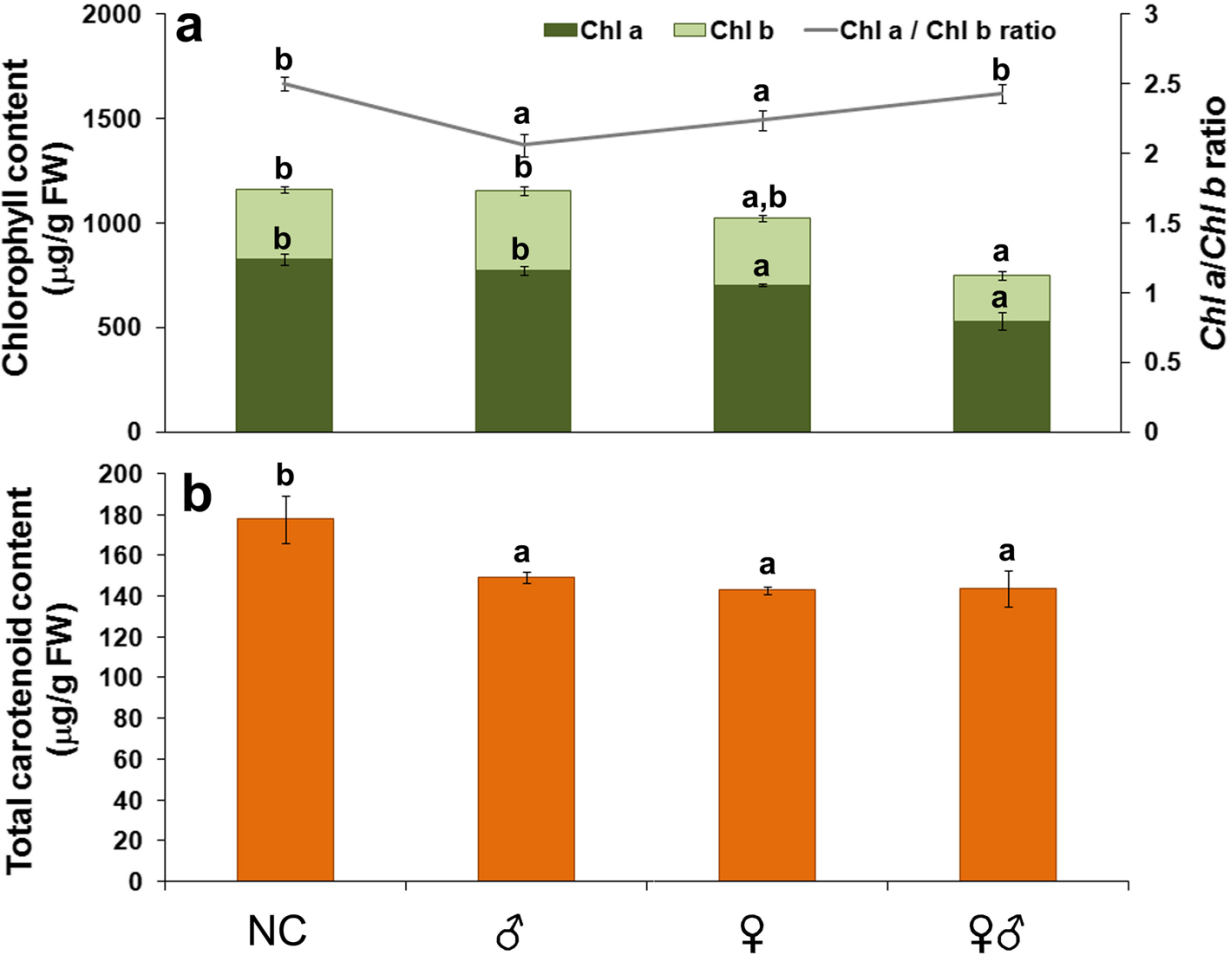
Effects of insect gender on photosynthetic pigments content in carrot leaves. **a** Total chlorophyll (*Chl a*, *Chl b*) and *Chl a/b* ratio; **b **Total carotenoid content in carrot leaves after one-hour infestation with male (♂), female (♀), or a mixture of male and female (♂♀) *B. trigonica*. NC—negative control, non-infested carrot plants. Data presented are means ± standard errors. Different letters indicate statistically significant differences according to LSD test (*p* ≤ 0.05)

One hour after insect infestation, photosynthetic pigment content in carrot leaves is affected by insect feeding (Fig. 1). Infestation with mixture of male and female individuals together caused the remarkably decreased the content of both analyzed chlorophylls (Fig. 1a). There were significant differences between female and male feeding in the chlorophyll (*Chl*) content of carrot leaves. In particular, a significant difference was observed in *Chl a* content in carrot leaves infested only with females in comparison to control and plants infested only with males (Fig. 1a).

Decrease in *Chl a* content (14.87%) was also detected in leaves of plants infested with female *B. trigonica* compared to the negative control, i.e. non-infested plants (NC). On the other hand, there was no significant difference in *Chl* content in carrot plants infested with male *B. trigonica* compared to control plants. The *Chl a/b* ratio in carrot plants infested with female or male decreased by 10.4 and 17.6%, respectively, compared to NC and plants infested with insects of both genders.

Total carotenoid content in carrot leaves was reduced one hour after *B. trigonica* infestation with no significant differences between insect sexes (Fig. 1b). Infestation with male (15.99%) or female (19.62%) or both sexes together (19.14%) resulted in a significant reduction of total carotenoid content in carrot leaves in comparison to non-infested-NC plants (Fig. 1b).

#### Oxidative stress response of carrot to different gender of *B*. *trigonica*

**Fig. 2:**
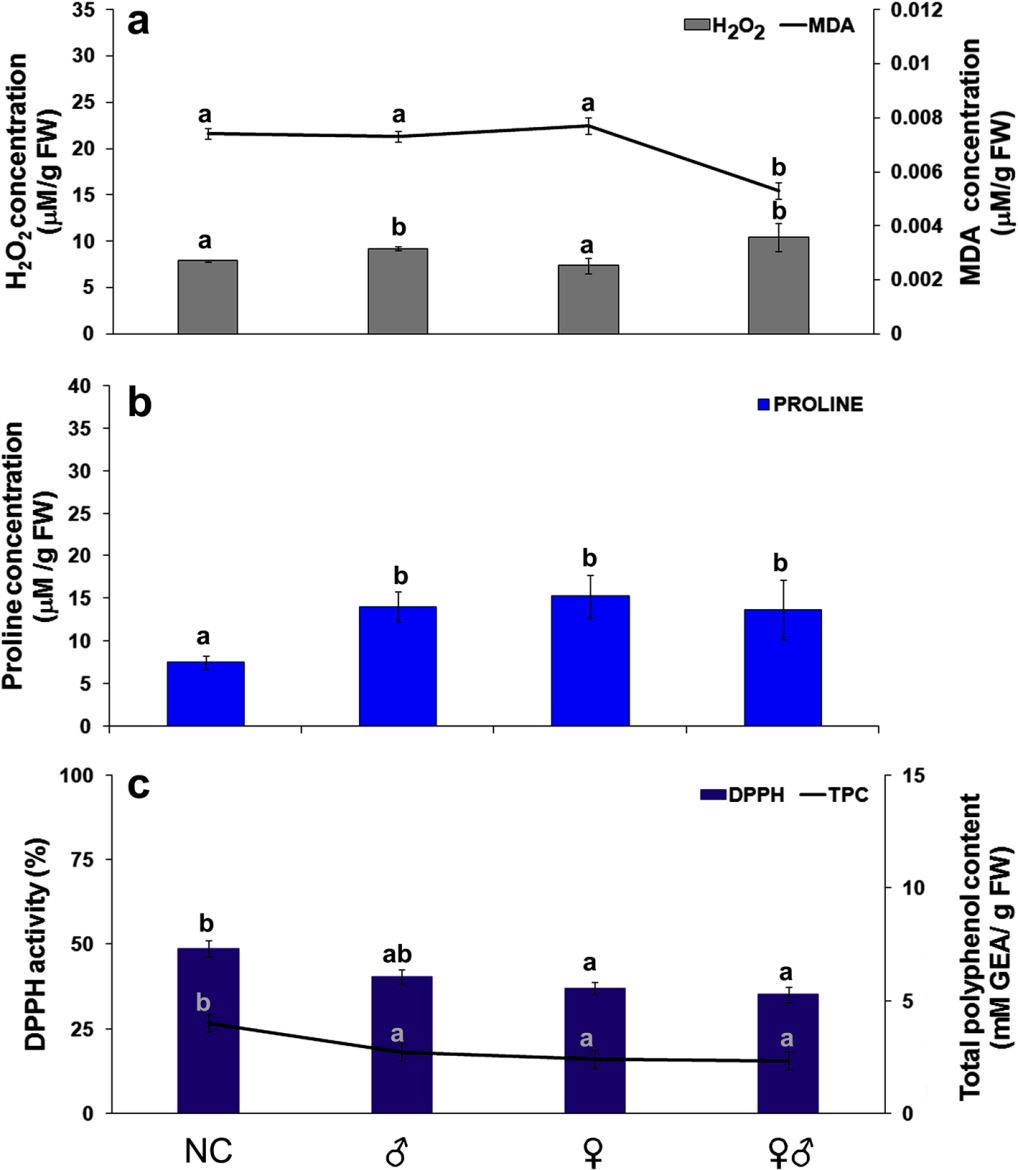
Effects of insect sex on oxidative stress indicators in carrot leaves. **a** H_2_O_2_ and MDA content; **b** proline content; **c** TPC and DPPH activity in carrot leaves after male (♂), female (♀), or mixture of male and female (♂♀) *B. trigonica* infestation. NC—negative control, non-infested carrot plants. Data presented are means ± standard errors. Different letters indicate statistically significant differences according to LSD test (*p* ≤ 0.05)

Oxidative stress indicators (H_2_O_2_, MDA, proline, TPC, and DPPH activity) in carrot leaves were also affected by infestation with male, female only, or a male and female together after one hour (Fig. 2). Infestation with *B. trigonica* male alone or with mixture of male and female increased H_2_O_2_ content (by 17.04% and 36.38%, respectively) in comparison to control plants. Interestingly, the H_2_O_2_ content of plants infested only with female did not change (Fig. 2a). In contrast to H_2_O_2_, a significant change in MDA content was observed only when both insect infested carrot plants compared to control and plants infested with insects of the same gender (Fig. 2a).

**Fig. 3:**
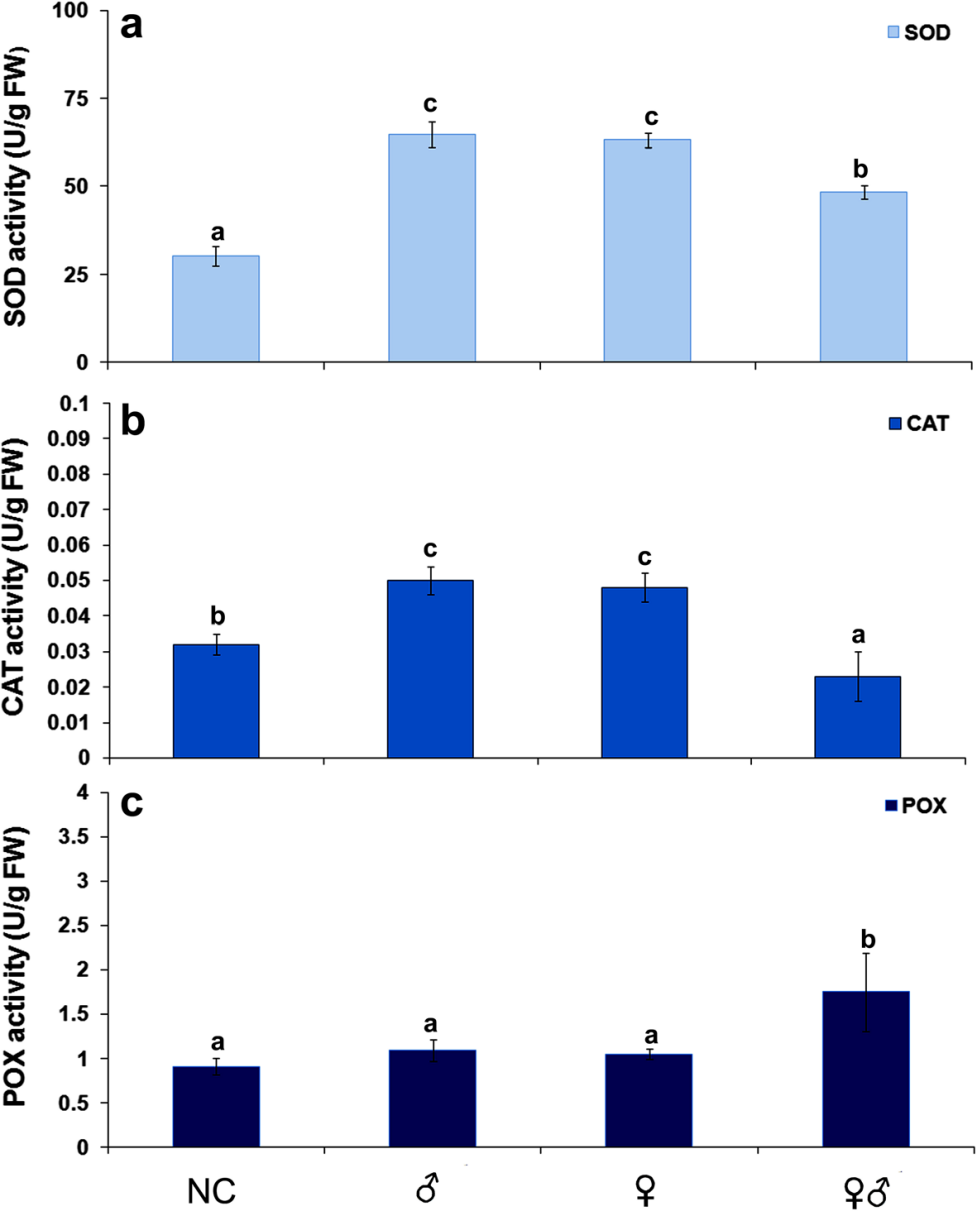
Effects of insect gender on antioxidative enzyme activities in carrot leaves. **a** SOD activity; **b** CAT activity; and **c** POX activity in carrot leaves after infestation with male (♂), female (♀), and mixture of male and female (♂♀) *B. trigonica*. NC—Negative control, non-infested carrot plants. Data presented are means ± standard errors. Different letters indicate statistically significant differences according to LSD test (*p* ≤ 0.05)

Proline content in carrot leaves was significantly increased (82.18–102.92%) when infested with *B. trigonica*, with no significant differences between insect genders (Fig. 2b). In contrast to proline, TPC was significantly decreased after *B. trigonica* infestation (17.91–39.80%) but without notably differences between insect sexes (Fig. 2c). Insect sex also affected the DPPH activity of carrot leaves. Female infestation caused significantly lower DPPH activity compared to male infestation and control plants (Fig. 3c). DPPH activity was also significantly lower in carrot plants infested by *B. trigonica* individuals of both genders.

In addition, insect feeding during one hour had a differential effect on antioxidative enzyme activities. SOD (Fig. 3a) and CAT (Fig. 3b) activities in carrot leaves were increased after infestation with male or female *B. trigonica* but decreased after infestation with both insect sexes one hour of inoculation. In general, there were no significant differences between separate infestations of male and female insects on antioxidative enzyme activities in carrot plants. In contrast to separate infestations, both male and female infestations caused a significant reduction of SOD activity (approximately 25%). Similarly, the activity of CAT was also reduced by 28.12%. On the other hand, the activity of POX was not significantly different from the activity of control plants when separate insect sexes were infested, but the activity of POX was increased by over 90% after infestation with both insect sexes (Fig. 3c).

### Physiological and oxidative stress response of carrot during the first six hours of *B*. *trigonica* infestation

#### Photosynthetic pigments content

**Fig. 4:**
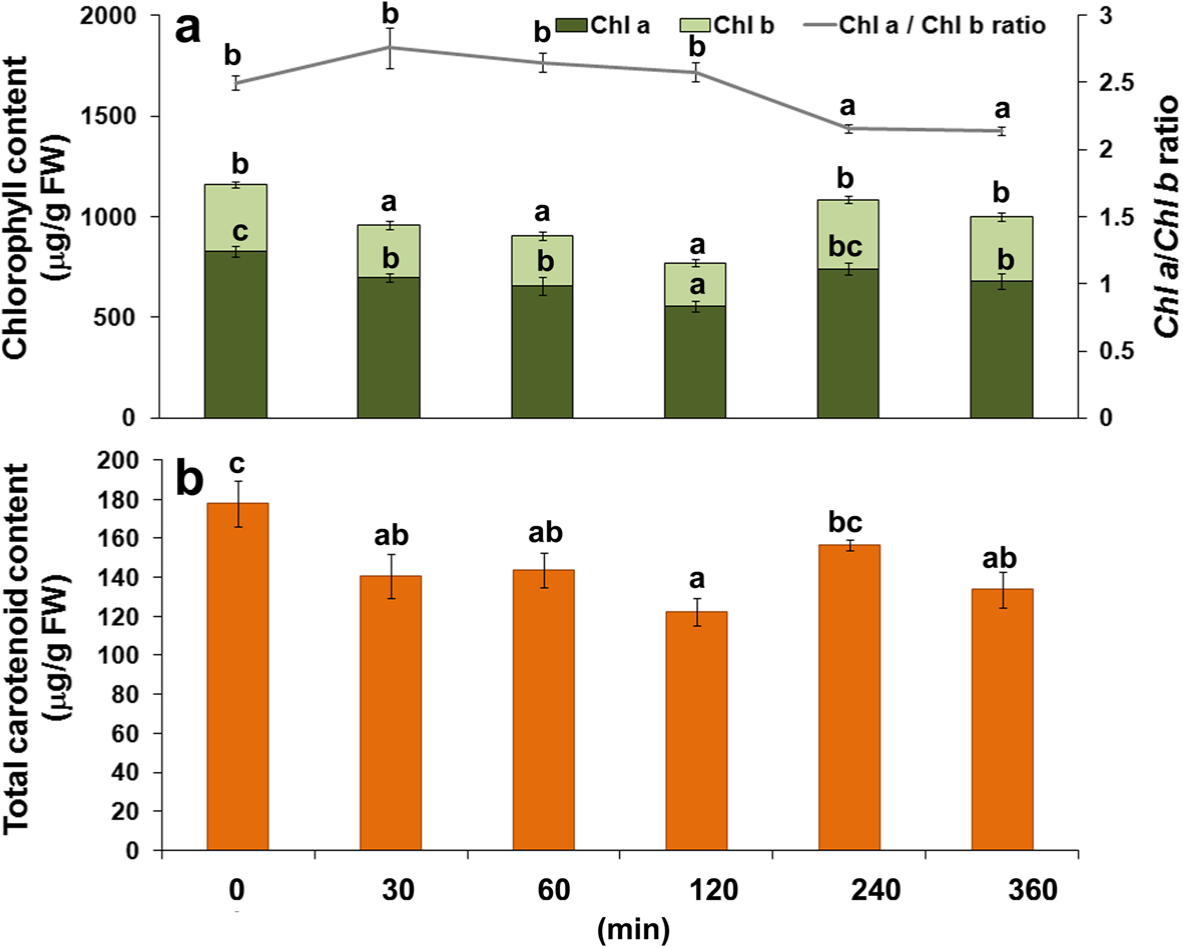
Photosynthetic pigment contents in carrot leaves during the first six hours after infestation with *B. trigonica*. **a** Total chlorophyll (*Chl a* + *Chl b*) and *Chl a/b* ratio; **b** Total carotenoid content. Data presented are means ± standard errors. Different letters indicate statistically significant differences according to LSD test (*p* ≤ 0.05)

Total *Chl* (*Chl a* + *Chl b*) and carotenoid content in carrot leaves steadily decreased during the first two hours after *B. trigonica* infestation with male and female together (Fig. 4). This initial decline ceased after four hours when *Chl* levels reached control levels (Fig. 4a). In addition, after four hours of infestation with *B. trigonica*, a significant change was observed in *Chl a/Chl b* ratio, where a marked decrement was observed. The total carotenoid content in the carrot leaves also decreased during the first two hours (Fig. 4b). At the end of the observation period, six hours after infestation with *B. trigonica*, the total *Chl* content was similar to control plants, while the total carotenoid content continued to reduce in comparison to control plants.

#### Oxidative stress response of carrot during the first six hours of *B*. *trigonica* infestation

**Fig. 5:**
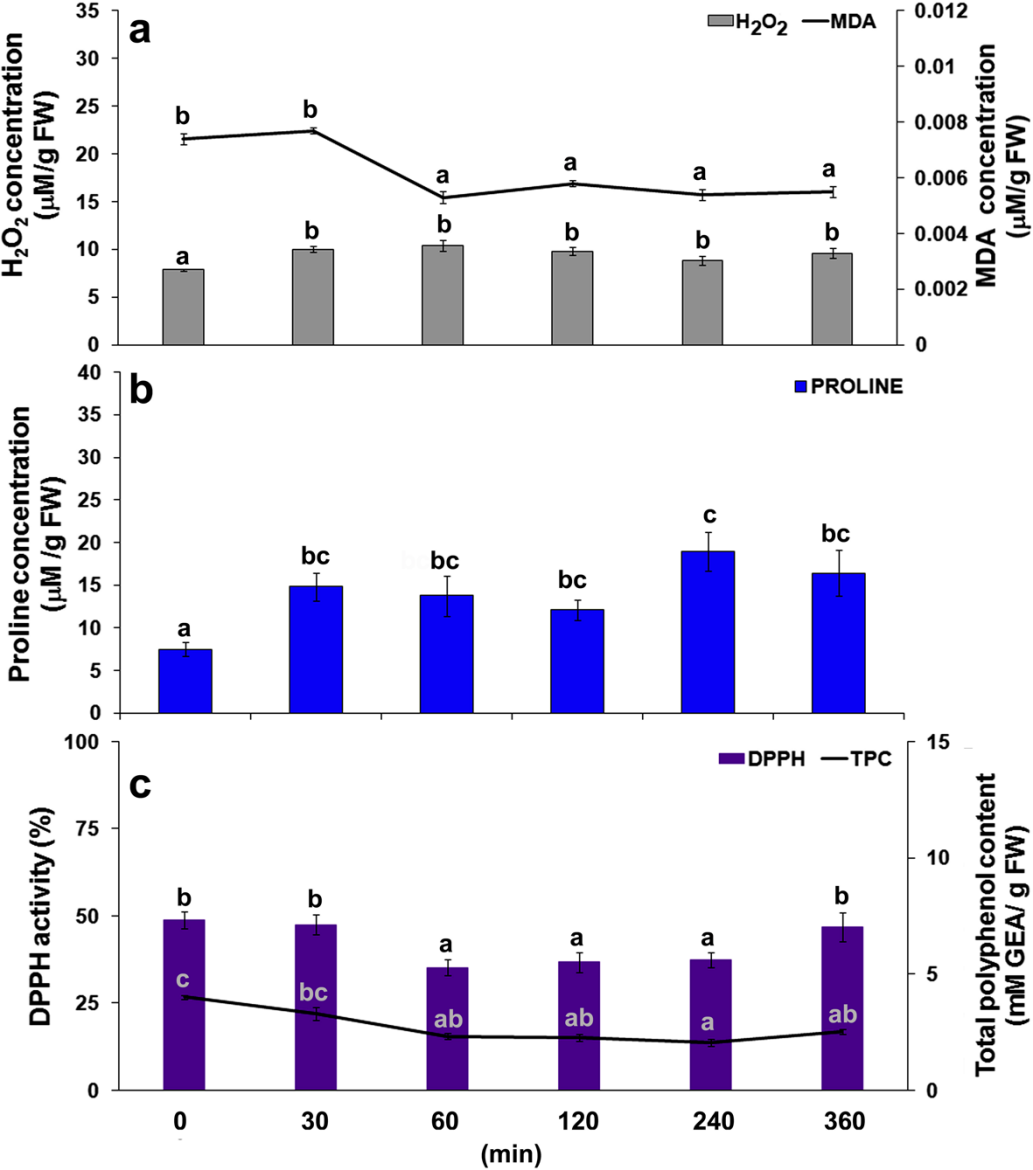
Indicators of oxidative stress in carrot leaves during the first six hours after *B. trigonica* infestation. **a** H_2_O_2_ and MDA content; **b** proline content; **c** DPPH activity and TPC. Data presented are means ± standard errors. Different letters indicate statistically significant differences according to LSD test (*p* ≤ 0.05)

Oxidative stress indicators in carrot leaves examined in the first six hours after *B. trigonica* infestation showed a significant change in response to insect infestation (Fig. 5). H_2_O_2_ content in carrot leaves was significantly elevated (13.74%) after 30 min and remained elevated up to six hours after *B. trigonica* infestation, with no significant difference in H_2_O_2_ content during this period (Fig. 5a). In contrast to H_2_O_2_ content, MDA content in carrot leaves was significantly decreased after *B. trigonica* infestation. The significantly decreased MDA content (28.37%) was observed one hour after inoculation of *B. trigonica* and remained at a similar level until the end of the study period (Fig. 5a).

Proline content in carrot leaves was significantly increased (above 97%) after only 30 min of infestation with *B. trigonica*, similar to H_2_O_2_ content (Fig. 5b). Thereafter, the increment of proline content in carrot leaves varied from 60.9 to over 152%, four hours after *B. trigonica* infestation, when the highest increase was recorded compared to the control. On the other hand, DPPH activity showed a similar pattern to MDA content (Fig. 5c). Decreased DPPH activity (27.67%) was observed one hour after *B. trigonica* infestation, except for the last time point (six hours), when DPPH activity was similar to control plants. In contrast to DPPH activity, TPC gradually decreased during the first six hours after the carrot plants were infested with *B. trigonica* (Fig. 5c).

**Fig. 6:**
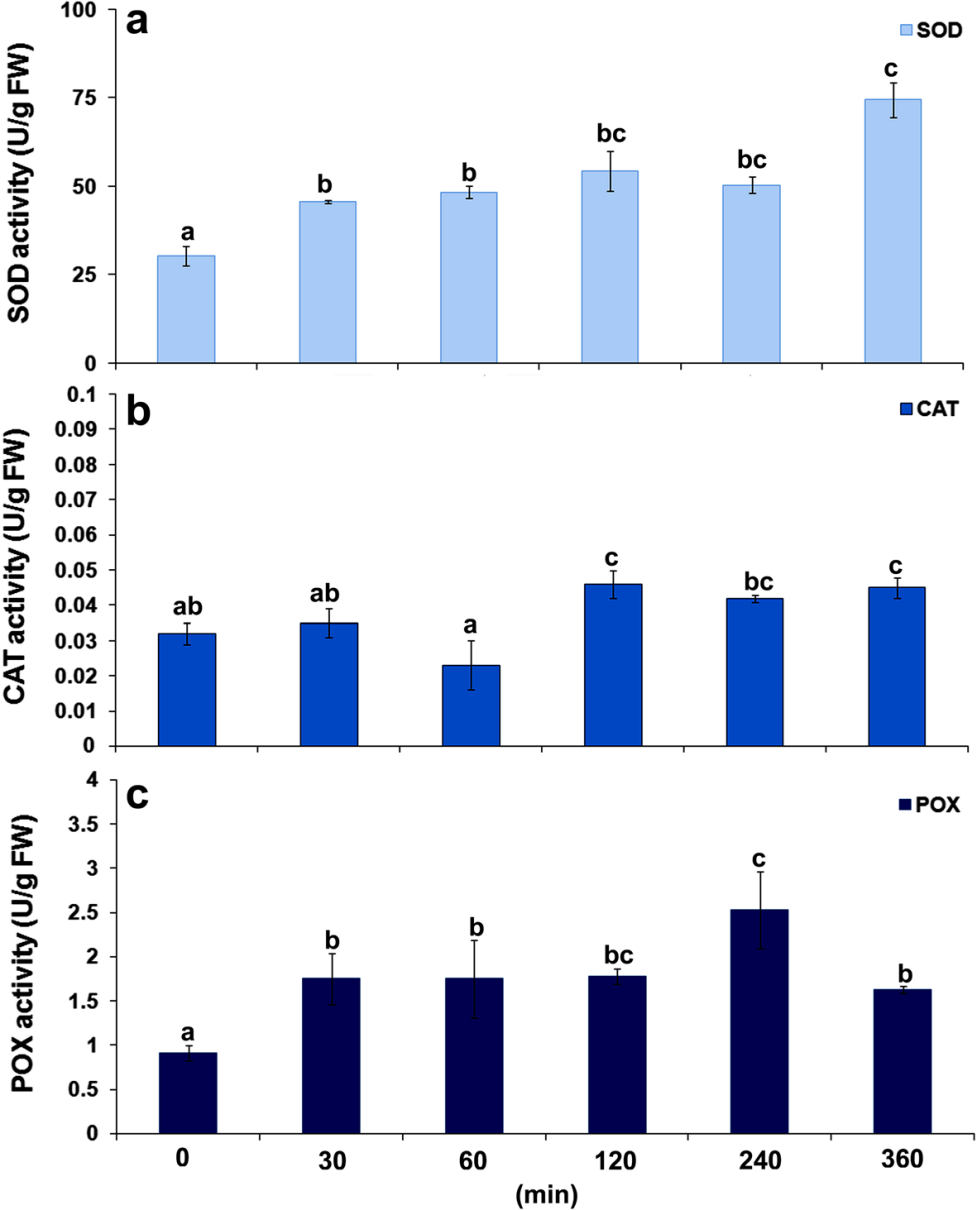
Antioxidative enzyme activities in carrot leaves during the first six hours of *B. trigonica* infestation. **a** SOD; **b** CAT; **c** POX activity. Data presented are means ± standard errors. Different letters indicate statistically significant differences according to LSD test (*p* ≤ 0.05)

The greatest change during the first six hours of *B. trigonica* infestation was observed in antioxidant enzyme activities (Fig. 6). After 30 min of *B. trigonica* infestation, superoxide dismutase activity was increased by more than 50% in carrot leaves. Thereafter, the activity of SOD gradually increased, and the highest value was reached six hours after infestation, when an increment of more than 146% was observed (Fig. 6a). CAT activity in carrot leaves decreased significantly one hour after *B. trigonica* infestation, and then a significant increment was observed (over 95%) (Fig. 6b). Like SOD activity, POX activity increased steadily during the first six hours of *B. trigonica* infestation. After 30 min, POX activity was 92.30% higher, while the highest activity (over 178%) was observed four hours after *B. trigonica* infestation in comparison to non-infected control plants (Fig. 6c).

### Physiological and oxidative stress response of carrot to long-term infestation with *B*. *trigonica*

#### Photosynthetic pigments content

**Fig. 7:**
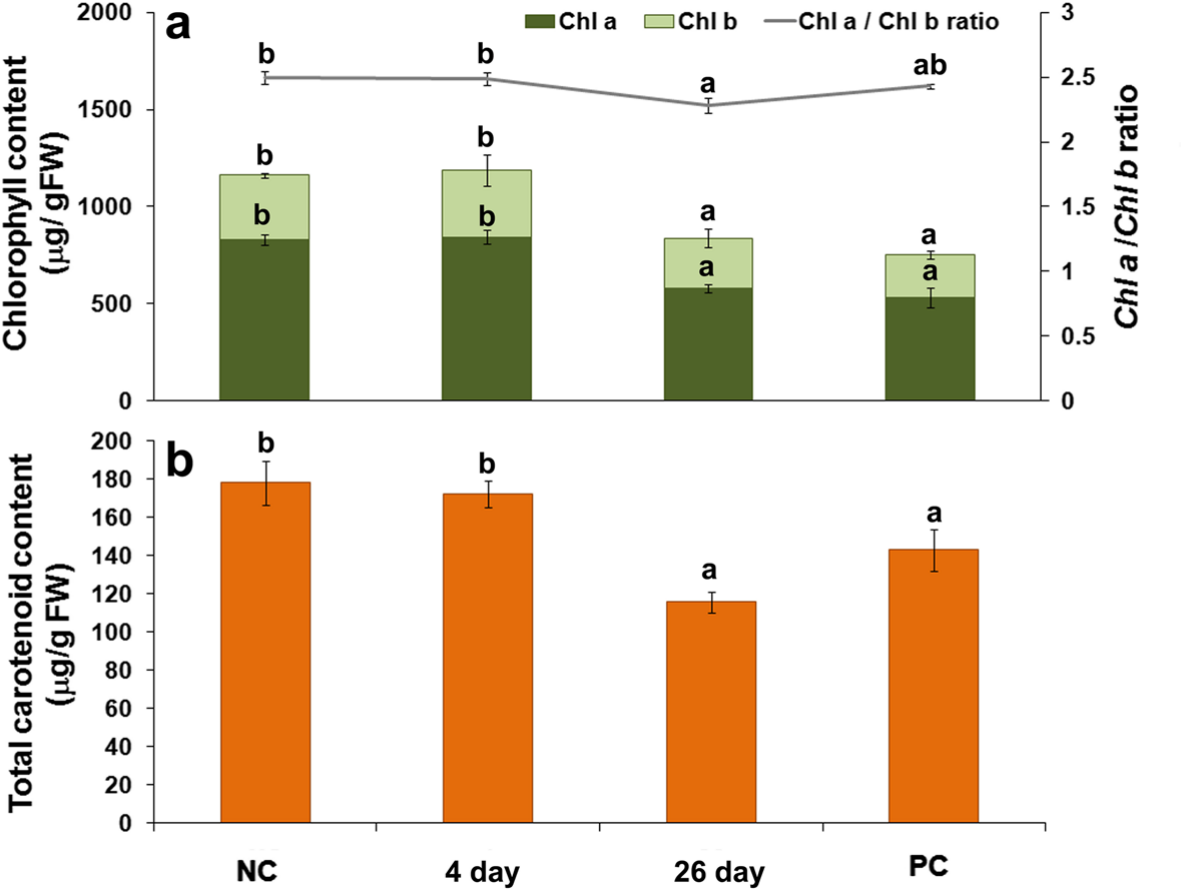
The effects of long-term *B. trigonica* infestation on photosynthetic pigments in carrot leaves. **a** Total chlorophyll (*Chl a*, *Chl b*) and *Chl a/b* ratio; **b** Total carotenoid content in carrot leaves after four and 26 days of *B. trigonica* infestation; NC – negative control, non-infested carrot plants; PC – positive control, parental carrot plants continuously exposed to insect infestation. Data presented are means ± standard errors. Different letters indicate statistically significant differences according to LSD test (*p* ≤ 0.05)

**Fig. 8:**
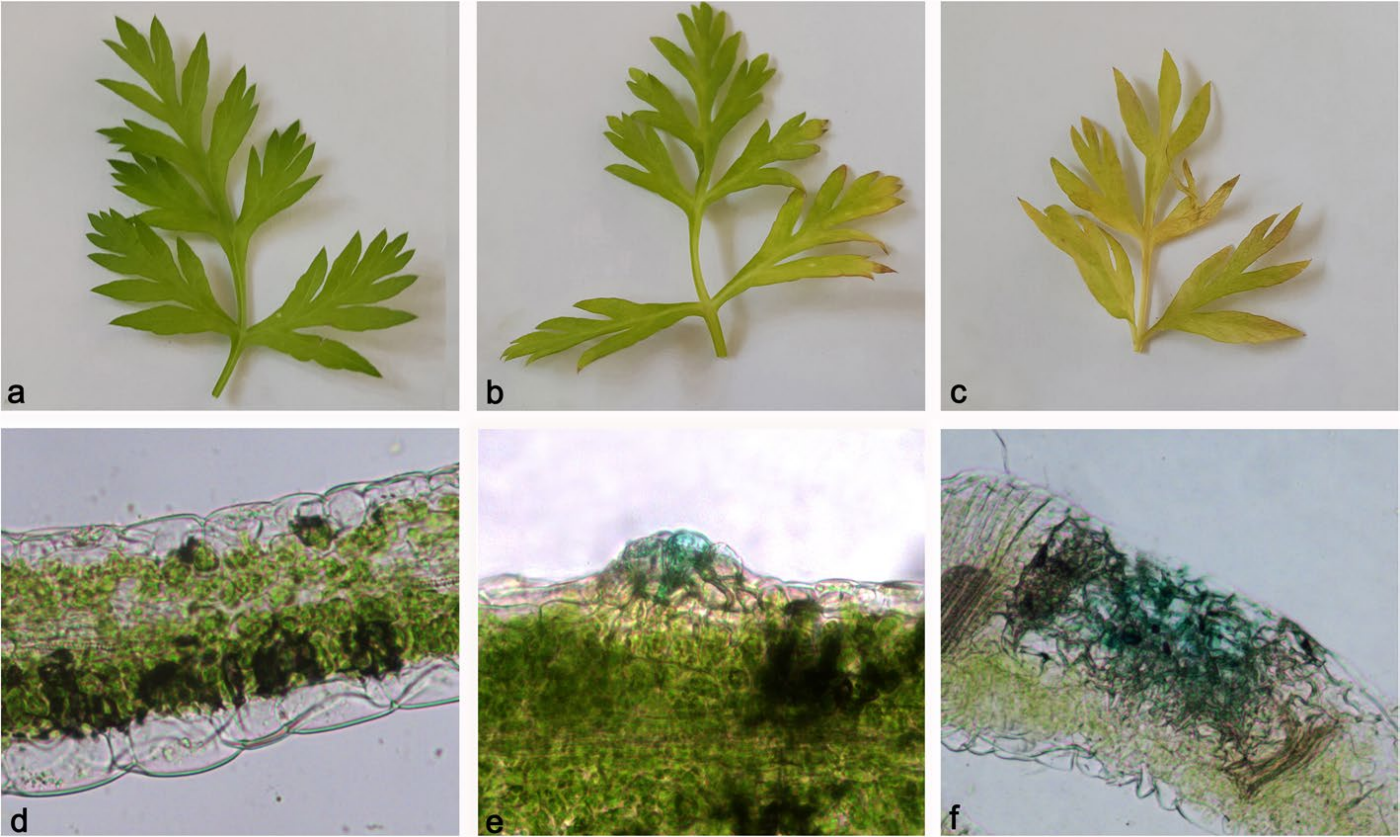
Morphology and vital staining of carrot leaves after infestation with *B. trigonica*. **a** leaf of a plant without insect infestation; **b** leaf of a plant after 26 days of infestation; **c** leaf of parental control plant; **d** cross section of a control leaf without insect infestation; **e** cross section of a leaf after 26 days of infestation; **f** cross section of a parental control leaf. *Note the blue-stained dead cells damaged by insect infestation, as evidenced by Evan’s blue staining (**e**, **f**)

A significant change in photosynthetic pigments content was observed in carrot leaves after long-term infestation with *B. trigonica* (Fig. 7). Total *Chl* content in carrot leaves, including *Chl a*, *Chl b*, and *Chl a/b* ratio, was significantly changed 26 days after inoculation with *B. trigonica* compared with control plants. Moreover, these changes were at the same level as in plants in the parental control that were continuously exposed to *B. trigonica* infestation (Fig. 7a). A significant degree of chlorosis and yellowing of carrot leaves was observed compared to the control plants (Fig. 8a, b). Total *Chl* content decreased by 27.83%, and accordingly, *Chl a*, *Chl b*, and *Chl a/b* ratio also decreased by 29.98, 22.66, and 8.80%, respectively, compared to the control plants (Fig. 7a). In addition to *Chl*, a significant decrease in carotenoid content (35.37%) was also observed in carrot leaves 26 days after inoculation with *B. trigonica* (Fig. 7b). The same level of total carotenoid content was also observed in the parental control plants (Fig. 7b), where the chlorosis of leaves was the most intense (Fig. 8c).

Compared to the control plants (Fig. 8a, d), massive cell damage due to insect feeding occurred 26 days after *B. trigonica* infestation, with dead cells visible (Fig. 8b, e). The largest leaf area with damaged and dead leaf tissue was observed in the parental plants that were constantly exposed to insect feeding (Fig. 8c, f).

#### Oxidative stress response of carrot to long-term infestation with *B*. *trigonica*

**Fig. 9:**
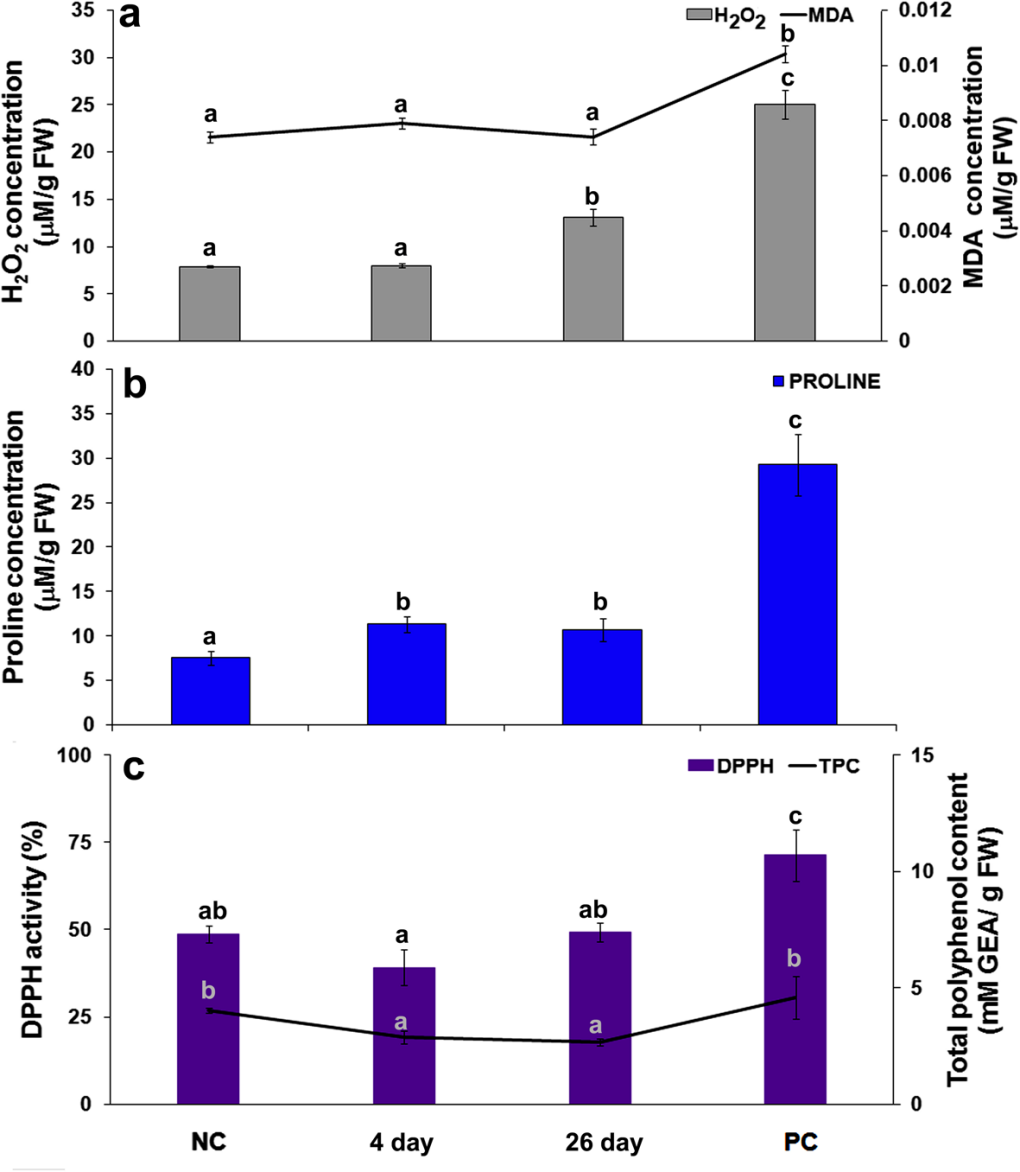
Indicators of oxidative stress in carrot leaves after long-term infestation with *B. trigonica*. **a** H_2_O_2_ and MDA content; **b** proline content; **c** TPC and DPPH activity in carrot leaves after 4 and 26 days of infestation. NC – negative control, non-infested carrot plants; PC – positive control, parental plants continuously exposed to insect infestation. Data presented are means ± standard errors. Different letters indicate statistically significant differences according to LSD test (*p* ≤ 0.05)

The studied oxidative stress indicators in carrot leaves showed a gradual increase in H_2_O_2_ and proline content in response to long-term infestation with *B. trigonica* (Fig. 9a, b). The increased H_2_O_2_ production observed during the first six hours of exposure (Fig. 6a) to *B. trigonica* levelled off after four days of infestation to the same level as in control plants (Fig. 9a). In response to 26 days of B. *trigonica* infestation, H_2_O_2_ production in carrot leaves increased by 66.15% compared to control plants. Parental plants continuously exposed to *B. trigonica* recorded the highest H_2_O_2_ production, which was 3.1 times higher than control plants (Fig. 9a).

**Fig. 10:**
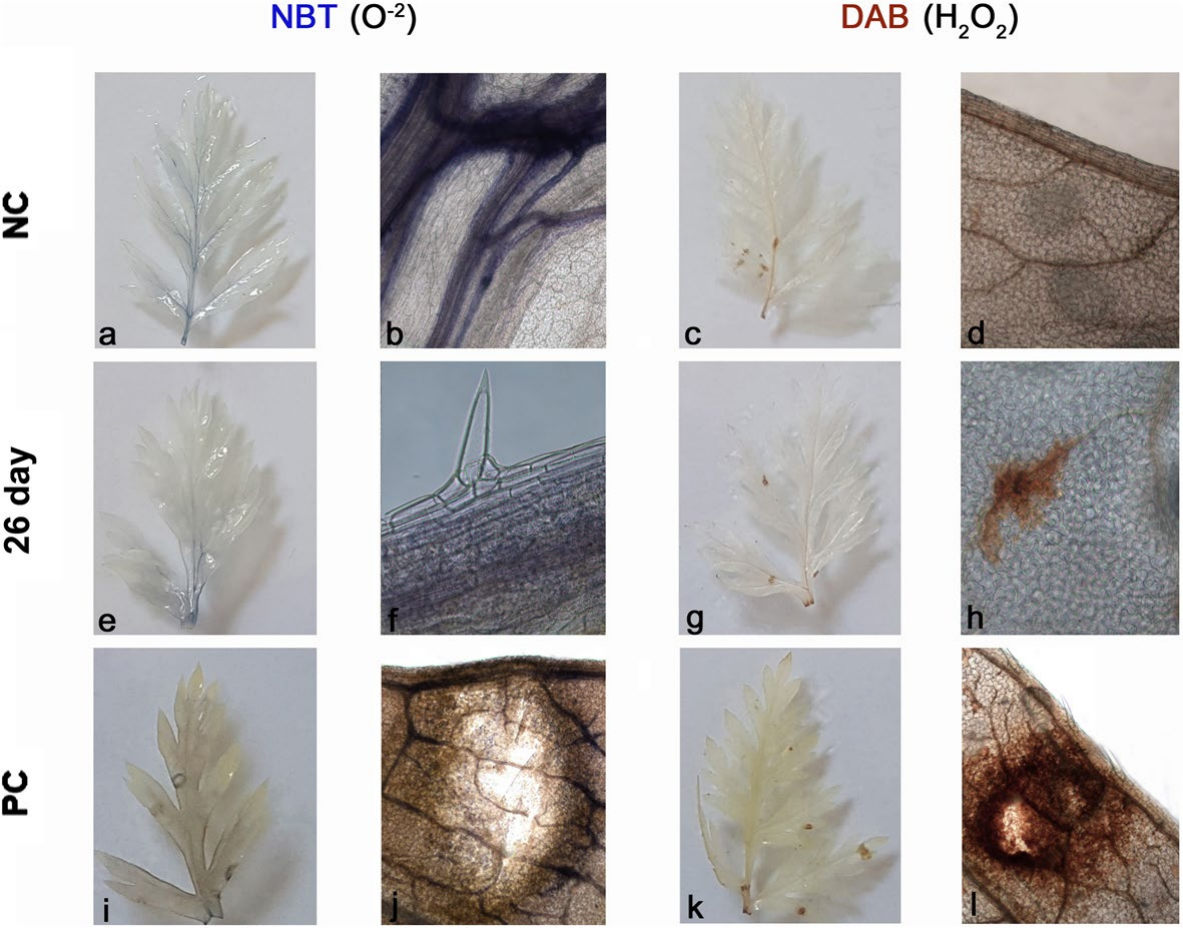
Histochemical localization of O_2_^−^ (NBT staining) and H_2_O_2_ (DAB staining) in carrot leaves after infestation with *B. trigonica*. **a**-**d** whole leaf and leaf section of negative control (NC), non-infested carrot plant following NBT (**a**, **b**) and DAB staining (**c**, **d**); **e**–**h** whole leaf and leaf section of carrot plants 26 days after *B. trigonica* infestation following NBT (**e**, **f**) and DAB staining (**g**, **h**); **i**-**l** whole leaf and leaf section of positive control, parental plants continuously exposed to insect infestation following NBT (**i**, **j**) and DAB staining (**k**, **l**). * Note: O_2_^−^ accumulation is colored blue, H_2_O_2_ accumulation is colored brown

To localize the production of ROS (O_2_^−^ and H_2_O_2_) in leaf tissue, we stained control, parental, and leaves of plants after 26 days of *B. trigonica* infestation with specific dyes (Fig. 10). In control plants, production of ROS (O_2_^−^ and H_2_O_2_) was mainly found in leaf veins (Fig. 10a-d). After 26 days of *B. trigonica* infestation, there was significant production of O_2_^−^ also in the leaf veins (Fig. 10f), and H_2_O_2_ accumulation at the sites where the insects were feeding intensively (Fig. 10h). The massive damage to leaf tissue caused by *B. trigonica* feeding is clearly visible on the parental plants (Fig. 10i-l). Significant accumulation of O_2_^−^ (Fig. 10j) and H_2_O_2_ (Fig. 10l) was observed where the insects caused cracks in the leaves by intensive feeding.

In contrast to H_2_O_2_, there were no statistically significant differences in MDA content after four and 26 days of *B. trigonica* infestation compared to control carrot plants, while significantly higher MDA content was observed only in the parental plants (Fig. 9a).

Proline content in carrot leaves remained significantly elevated (over 50%) even after four days of *B. trigonica* infestation in comparison to control plants (Fig. 2b). Proline content remained at similar levels after 26 days of infestation, while the highest value was recorded in the parent plants, which was 3.8 times higher than in the control plants (Fig. 9b), as in case of to H_2_O_2_ and MDA content (Fig. 9a). TPC and DPPH activity in carrot leaves decreased by 28.11 and 19.17%, respectively, four days after inoculation with *B. trigonica* compared to control plants (Fig. 9c). After 26 days of infestation with *B. trigonica*, the TPC and DPPH activity continued to reduce, while the TPC in the parental plants reached the level similar to control plants. DPPH activity was significantly increased in the control parental plants, reaching the highest activity, 46% more than in the control plants (Fig. 9c).

**Fig. 11:**
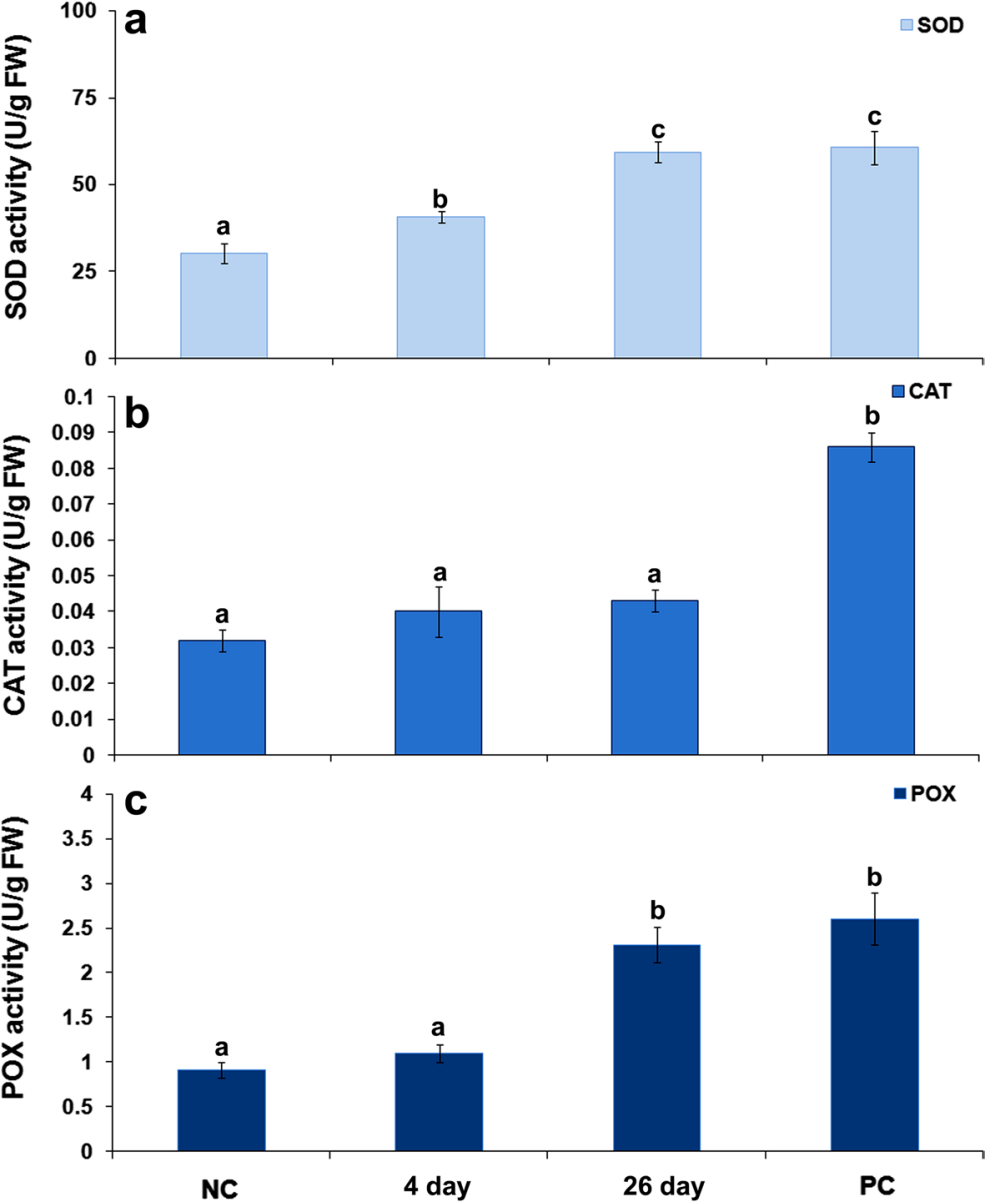
Antioxidative enzyme activities in carrot leaves during long-term *B. trigonica* infestation. **a** SOD activity; **b** CAT activity, and **c** POX activity in carrot leaves after 4 and 26 days of infestation. NC – negative control, non-infested carrot plants; PC – positive control, parental plants continuously exposed to insect infestation. The data presented are mean value ± standard errors. Different letters indicate statistically significant differences according to LSD test (*p* ≤ 0.05)

In general, the activity of antioxidant enzymes in carrot leaves were increased by *B. trigonica* infestation, but with varying degrees and timing of increased activity (Fig. 11). Infestation with *B. trigonica* resulted in a gradual increase in SOD activity in carrot leaves. The SOD activity was 34.80% higher than in control plants after four days and 96% higher 26 days after inoculation (Fig. 11a). The similar SOD activity was observed in parental plants 26 days after inoculation with *B. trigonica*. In contrast to SOD, CAT activity remained at the same level as control plants throughout the exposure period. A similar level of CAT activity was observed after two hours (Fig. 6b) and after four and 26 days of *B. trigonica* infestation (Fig. 11b). The highest CAT activity was observed in the parent plants, which was 2.6 times higher than in the control plants (Fig. 11b). There was no increase in POX activity in carrot leaves in comparison to control plants four days after *B. trigonica* infestation. The significantly increased POX activity was observed 26 days after *B. trigonica* infestation and in parental plants. POX activity in these plants were 153.85 and 185.71% higher than the POX activity in control plants (Fig. 11c).

## Discussion

Successful of plants in resisting biotic stress caused by insects, depends on their ability to quickly recognize and decode the incoming signal and respond appropriately to the insect infestation. These initial changes can occur within a few hours to several days or even weeks after insect attack. To investigate the mechanism of carrot defense to jumping plant-louse psyllid, we infested carrots with *B. trigonica* under controlled laboratory conditions. The physiological and oxidative stress defense responses of carrots to *B. trigonica* infestation within the first six hours after insect infestation and during establishment of the first generation were evaluated. This approach enable focus of this research on changes related to plant–insect interaction and minimize the influence of all other factors that may interfere with other types of stress that may occur in the field. All changes in physiological responses and non-enzymatic and enzymatic components of oxidative stress responses are discussed separately.

### Physiological response of carrot to *B*. *trigonica* infestation

Plant response to insects involves reprogramming of plant physiology and requires some degree of interaction, particularly between primary metabolism, photosynthesis, and secondary metabolism of host plants [30, 46, 48–50]. The photosynthetic pigments content may be one of the most important physiological parameters of primary metabolism affecting host plant–insect interactions [51, 52]. In this study, we found a significant decrease in photosynthetic pigments in carrot leaves due to *B. trigonica* infestation. Changes in photosynthetic pigments can be useful in studying plant resistance mechanisms and allow the use of photosynthetic pigments as markers to identify the tolerance of plant species to insect feeding. Numerous studies have documented an overall reduction in total chlorophyll and carotenoids in susceptible plants in response to insect feeding, while increased photosynthetic activity has been reported in many examples of plant tolerance to insects [53–59]. According to our results, *Chl* content in carrot leaves was significantly affected by feeding insects of different genders. The most significant change in *Chl* content was observed after feeding female insects one hour after infestation. The observed differences could be related to the frequency and duration of feeding by females. Females of *B. trigonica* ingested longer from phloem sieve elements and reached phloem tissue more frequently than males [7]. The increased phloem ingestion of females compared to males could be explained by their need for a greater amount of nutrients for egg production. Female psyllids are capable of laying up to 900 eggs during their lifetime and require a large amount of nutrients to produce such a large number of eggs. In addition, there is much evidence that insect feeding can have differential effects on *Chl a* and *Chl b* content, as we found in *B. trigonica* [60–63]. All of our results considering decreased in photosynthetic pigment content are in accordance with other studies on psyllid species and other Hemipterans [6, 64–66]. In addition, we found that the combined infestation of males and females caused the highest decrease in total *Chl* content in carrots. This decrease could be related to the higher total number of *B. trigonica* individuals inoculated on carrots, as well as the greater probing of plant tissue by the insects.

Carotenoid content in carrot leaves was also reduced by *B. trigonica* infestation, but not caused by the different insect sex. According to our results, *B. trigonica* feeding caused a significant gradual decrease of both photosynthetic pigments content, which started after 30 min and lasted for two hours, without any change in the *Chl a*/*Chl b* ratio. A significant initial decrease in *Chl* and carotenoid content in response to herbivorous insects has also been reported for many plant species [63, 67]. According to our results, increased level of photosynthetic pigments in carrot leaves was observed four hours after infestation with *B. trigonica*. This increase amplified the level of pigment content in infested plants without any visible change in leaf color and persisted for four days after *B. trigonica* infestation. These results support the hypothesis that phloem-feeding insects such as *B. trigonica* can increase photosynthetic rates of host plants [68, 69]. This increased photosynthetic intensity contributed to increased metabolism and delayed initiation of plant defense responses in plants, allowing plants to tolerate the presence of pests and remained in good condition for some time [30, 46, 70]. We observed significant damage only during nymphal and imago feeding (26 days) and not during the period when *B. trigonica* females laid eggs that began to hatch on carrot leaves (four days). These results could be explained by the increased feeding of *B. trigonica* nymphs and imago on carrot plants, which leads to visible symptoms [19]. In addition, previous studies have shown that psyllids significantly change the color of carrot leaves [5–7]. The observed leaf yellowing is a first visible symptom of induced senescence caused by wounding of the susceptible plant by insect attack [71]. Photosynthetic pigments content in carrot leaves of field-collected carrot plants with the same symptoms gradually decreased with increasing insect density on the plants, whereas Chl a was not detected in leaves of carrot plants infested by 15 nymphs [4]. Leaf yellowing due to insect attack differs from natural leaf senescence, in which chloroplasts remain intact and apparently functional, whereas leaf yellowing caused by psyllids has been observed to involve lysis of thylakoid membranes of chloroplasts in mesophyll cells due to the action of salivary enzymes [71, 72]. Phloem-sucking insects such as *B. trigonica* elicit more specific responses, as the withdrawal of phloem and xylem contents disrupts both the water and nutrient budgets of the plant and effectively modulates chloroplast functions. We have observed that *B. trigonica* causes significant damage to carrots by injecting toxic saliva during feeding as well as by directly damaging the palisade tissue containing the chloroplast. Similar results have been described for many other plants in response to insect attack, such as tomato (*Solanum lycopersicum*) to melon and cotton aphid (*Aphid gossipii*) [31], fava bean (*Vicia faba*) to storage insect pests (*Callosobruchus* sp., *Acanthoscelides* sp., and *Bruchus* sp.) [73], mulberry (*Morus* sp.) to mealy bugs (*Moconellicoccus hirsutus, Paracoccus marginatus*) [74], and maize (*Zea mays*) to pink stem borer (*Sesamia inferens*) [75].

### Oxidative stress response of carrot to *B*. *trigonica* infestation

In response to insect feeding, ROS have been identified as early signals that integrate and regulate stress tolerance [46, 76, 77]. Plants respond to these signals with complex constitutive and inducible defense mechanisms such as synthesis of different types of compounds and activation of key defense enzymes. Only few studies measured ROS within the first hours after insect attack [78, 79]. According to our results, feeding on *B. trigonica* resulted in a significant increase of H_2_O_2_ production in carrot leaves. H_2_O_2_ content increased 30 min after insect attack and reminded at similar high level for several hours, and then decreased back to baseline level as in control plants. This baseline level of H_2_O_2_ production was observed during the egg-laying phase (four days), and another peak was observed during the nymphal and imago phases (26 days) of *B. trigonica* feeding. Some plants can produce ROS in response to insect eggs, effectively combating future larval herbivory [47], which is not the case for carrots in response to *B. trigonica* eggs. The carrot plants that were constantly exposed to insects produced the highest H_2_O_2_ content. It is very well known that H_2_O_2_ production in plant tissues increases as long as infestation continues [80]. The first sign of insect feeding on the plant produces an electrical signal at the site of damage (wound) that spreads to the entire leaf and leads to changes in the electrostatic membrane potential, producing ROS and Ca^2+^ as secondary messengers [81]. Second, insects excrete toxic saliva containing glucose oxidase, which triggers a plant defense response and production of H_2_O_2_ [81–84]. In our study, we found that H_2_O_2_ in carrot leaves exhibited a biphasic accumulation pattern similar to the oxidative burst induced by many other insects in different plants [79, 85–87]. It is postulated that early in life and at low levels of infestation, cell walls (apoplast) are the main sites where H_2_O_2_ is produced, while later, both extracellular and intracellular sources of ROS contribute to the oxidative burst in response to insect infestation [88]. In our study, male individuals of *B. trigonica* induced higher H_2_O_2_ production in carrot leaves compared to females. These results support the hypothesis that the response of plants to insect attack, especially in the initial phase of insect infestation, involves not only wounding but also sex pheromones [89]. In contrast to H_2_O_2_, we recorded a significant decrease in MDA one hour after insect feeding, which remained at a low level for the first six hours and then increased to the level of non-infested carrot plants. Also, there were also no differences in MDA content in carrot leaves associated with feeding insects of different genders. The final product of lipid peroxidation, MDA, as marker of oxidative stress, was significantly increased only in carrot plants continuously exposed to *B. trigonica* infestation. This is consistent with our findings that oxidative stress induced by constant feeding by *B. trigonica* alters cell membrane properties and induces cell death of carrot leaf cells.

Significantly increased proline accumulation in carrot was observed in response to insect infestation. Increased proline content occurred 30 min after *B. trigonica* infestation and remained at a high level throughout the whole study period. Proline, as a universal osmolyte, is part of the plant defense response that accumulates in plants in response to various stresses [90, 91]. Increased proline content during insect attack and stress in general serves as a source of energy and ROS scavenger [41, 92]. In addition, proline is a good marker of drought stress in plants caused by continuously removing of assimilates by phloem-sucking insects [93]. According to our studies, proline accumulation in carrot tissue can be used as a marker to determine the extent damage of carrot caused by *B. trigonica* infestation, as recently suggested by Ben Othmen et al. [4].

Plants can produce a large number and variety of organic compounds in their secondary metabolism that play an important role in direct or induced responses to herbivore attack [46]. These compounds can reduce the nutritional value of plant foods or act as deterrents or toxins to insects [36]. One of the large group of secondary metabolites is plant phenolics, which are the most common and widespread group of defense compounds that generally play an important role in host plant resistance to insects. Insect feeding can lead to tremendous changes of phenolic compounds [4, 94, 95]. These changes are not consistent, with some studies reporting higher while others reporting a reduced TPC. In addition, there are scarce literature data considering the dynamics and timing of changes in TPC due to insect feeding [95]. According to our results, *B. trigonica* feeding led to a significant decrease in TPC content in carrot leaves. This change began one hour after infestation and remained low throughout the whole study period. TCP content also decreased after both females and males feeding. These results are consistent with previous studies describing that the type of insect feeding can significantly affect the TPC of plants after insect infestation [93, 96]. In general, insects that fed by sucking decreased the TPC of the host plant, whereas chewing insects generally increased the TPC of the host plants [95]. Sucking insects such as *B. trigonica* used their salivary sheaths to suppress or inhibit the initiation of host defense responses. These putative defense suppressors (called effectors) have been described primarily in phloem feeders [97]. They cause severe damage by depriving plant nutrients or injecting plant elicitors or pathogens [7, 34]. This is an evolutionary strategy that allows these insects to feed long-term on living plant tissues and provide homeostasis to plant physiology for some time. In contrast to our results, a positive correlation between the density of *B. trigonica* and the increase in TPC content was observed in carrot plants collected in the field [4]. The greater increase in phenolic content observed, in field-grown carrot plants, occurred after long-term insect feeding and was the result of significant plant damage, which subsequently led to plant cell death [4, 95]. Our studies showed that *B. trigonica* feeding continuously alters the antioxidant properties of many other secondary metabolites in carrot leaves. We found that the antioxidant DPPH capacity of carrot leaves decreased during the first two hours after *B. trigonica* infestation, while a significant increase was observed only after prolonged insect infestation. These changes in DPPH activity, i.e., antioxidant capacity of secondary metabolites, indicate significant changes in the oxidative status of carrot due to long-term insect infestation.

### Antioxidative enzymes response of carrot to *B*. *trigonica* infestation

Oxidative burst upon insect attack is one element of plant defense mechanism, while on the other hand, tolerance to high ROS is the most important factor affecting plant growth and development under biotic stress [81, 98, 99]. In response to insect attack, plants activate a variety of defense mechanisms to defend against insect attack, including activation of important antioxidant enzymes. Our study showed an increase in SOD activity in all infested carrot plants compared to non-infested plants. SOD enzyme is unique in regulation of H_2_O_2_ and O_2_ concentration, which is central defense mechanism since H_2_O_2_ acts as a signaling molecule in the interactions between the plant and insects [44, 100, 101]. Similarly, SOD represent the first line of the defense in carrot against ROS produced by *B. trigonica* attack in carrot. SOD activity was induced 30 min after insect attack and gradually increased during first six hours and further during whole examined period. These enzyme converts superoxide anion radical to H_2_O_2_ and water, and it is found in numerous subcellular compartments [102]. Increased SOD enzymatic activity in carrot is associated with feeding injury by the insect. Further, ROS-detoxifying enzymes, CAT and POX convert H_2_O_2_ to water and oxygen. In general, the components of the antioxidant defense system act synergistically to perform ROS detoxification, but their activities are not the same and may also remain unchanged or even decrease, depending on the developmental stage, environmental stimuli, and the need to remove the ROS produced in the cells [103].

In addition to SOD, the activity of CAT in carrot leaves was also altered by *B. trigonica* infestation. The activity of CAT decreased significantly in the first hour after *B. trigonica* infestation, while a significant increase was observed 2–6 h after insect infestation. Furthermore, balanced CAT activity in carrot leaves was observed throughout the period of oviposition and nymphal feeding of *B. trigonica*. We proposed that the observed balanced activity of this hydrogen peroxide-degrading enzyme was apparently specifically triggered by the eggs or the oviducts associated with the eggs, as has been reported for some other laid eggs of herbivorous insects [47]. Increased CAT activity has often been associated with plant defense against infestation by chewing insects [43, 104]. Furthermore, increased CAT activity after insect feeding is known to increase plant resistance to insects [43]. However, in some species, insect feeding did not result in any changes in CAT activity [105, 106]. According to our results, reduced CAT activity in carrot leaves was observed one hour after *B. trigonica* infestation. This phenomenon of reduces CAT activity was also observed after aphid infestation in rice, wheat and sorghum [43, 107, 108]. Interestingly, the plant hormone salicylic acid (SA) is known to inhibit CAT in plants [47, 109]. In contrast to CAT, the activity of POX was increased in carrot leaves, being highest four hours after infestation and then decreasing. Our results suggested that SOD and POX are the most important enzymes involved in the defense response of carrot to *B. trigonica* infestation during the nymphal and adult generations of the insects.

Alterations in oxidative status and components of the antioxidant defense system have been described for many plant species [69, 93, 96, 110, 111]. Indeed, antioxidant enzyme responses differ in susceptible and resistant genotypes and plant species [61]. It was found that plant species susceptible to certain insect, such as carrot to *B. trigonica*, had lower CAT activity than control plants. There are several possible mechanisms by which insects can alter ROS levels in plants. The first studies of oxidative responses to phloem suckers assumed that they due to oxidases in the saliva of aphids facilitate the infestation process by detoxifying and mitigate plant defenses and altering plant growth [112, 113]. This hypothesis is supported by observations that phloem-sucking saliva contains peroxidases and other oxidizing enzymes [83, 113–115]. In addition, aqueous saliva can generate H_2_O_2_ in vitro when supplied with catechin as a substrate [82]. The present study summarizes that the significant increase in the activities of CAT, SOD, and POX was observed only in the damaged carrot leaves with increasing feeding duration, suggesting that the carrot plants are trying to defend themselves against long-term insect feeding. Our results show that a slight increase in SOD activity was observed during the oviposition of *B. trigonica*, whereas there were no changes in CAT and POX activities. We can assume that the H_2_O_2_ produced by SOD activity was mainly neutralized by the activity of the non-enzymatic component of the antioxidant defense system—proline. On this basis, it is evident that long-term exposure of carrot to *B. trigonica* infestation causes a higher level of oxidative stress, accompanied by induced activities of antioxidant enzymes, mainly SOD and POX. The production of H_2_O_2_ as a result of the high activity of SOD could be considered as a defense response of the plant to the insect attack, while on the other hand, the increased activity of POX could be related to the neutralization of excessively produced H_2_O_2_ to maintain the balance between production and degradation of ROS under stress. These results suggest that the enzymatic component of the antioxidant defense system is mainly responsible for the defense response of carrot to *B. trigonica* after long-term feeding.

## Conclusion

To our knowledge, this is the first comparative study documenting physiological and biochemical changes in carrot leaves as a result of *B. trigonica* infestation. The results of our study strongly suggest that *B. trigonica* infestation causes significant changes in primary and secondary metabolism and an attenuated ROS defense response in carrot leaves that allows long-term insect feeding. After *B. trigonica* feeding, lower levels of photosynthetic pigments, increased ROS and proline accumulation, delay of TPC accumulation and balanced CAT activity were observed in carrot leaves. The results suggest that the oxidative stress in carrot tissues triggered by *B. trigonica* infestation was mainly associated with increased activities of SOD and POX enzymes, after long-term infestation which are classified as the crucial antioxidant components in the response of carrot to *B. trigonica* infestation. Additional studies on molecular level are needed in order to complete the mechanisms of defense responses and may enable the use of these information in protection and sustainable carrot production.

## Materials and methods

### Plant cultivation and *B*. *trigonica* farming

The experiment was conducted from February to June 2021 at the Faculty of Agriculture, University of Belgrade. Seeds of *D. carota*_*,*_* cv.* Nantes (Seme Semena, Belgrade, Serbia) were used as initial plant material for the experiments. They were firstly sown in 5 × 5 cm pots, and the developed seedlings were transplanted into larger pots (10 cm) filled with the growing substrate for potted plants with 75% organic material (Agro CS, Hungary). Seeds germination and plants cultivation were performed in a growth chamber (GC-300TLH, Jeio Tech, Daejeon, Republic of Korea) under controlled conditions at a temperature of 22 ºC and a photoperiod of 16/8 h (day/night).

**Fig. 12:**
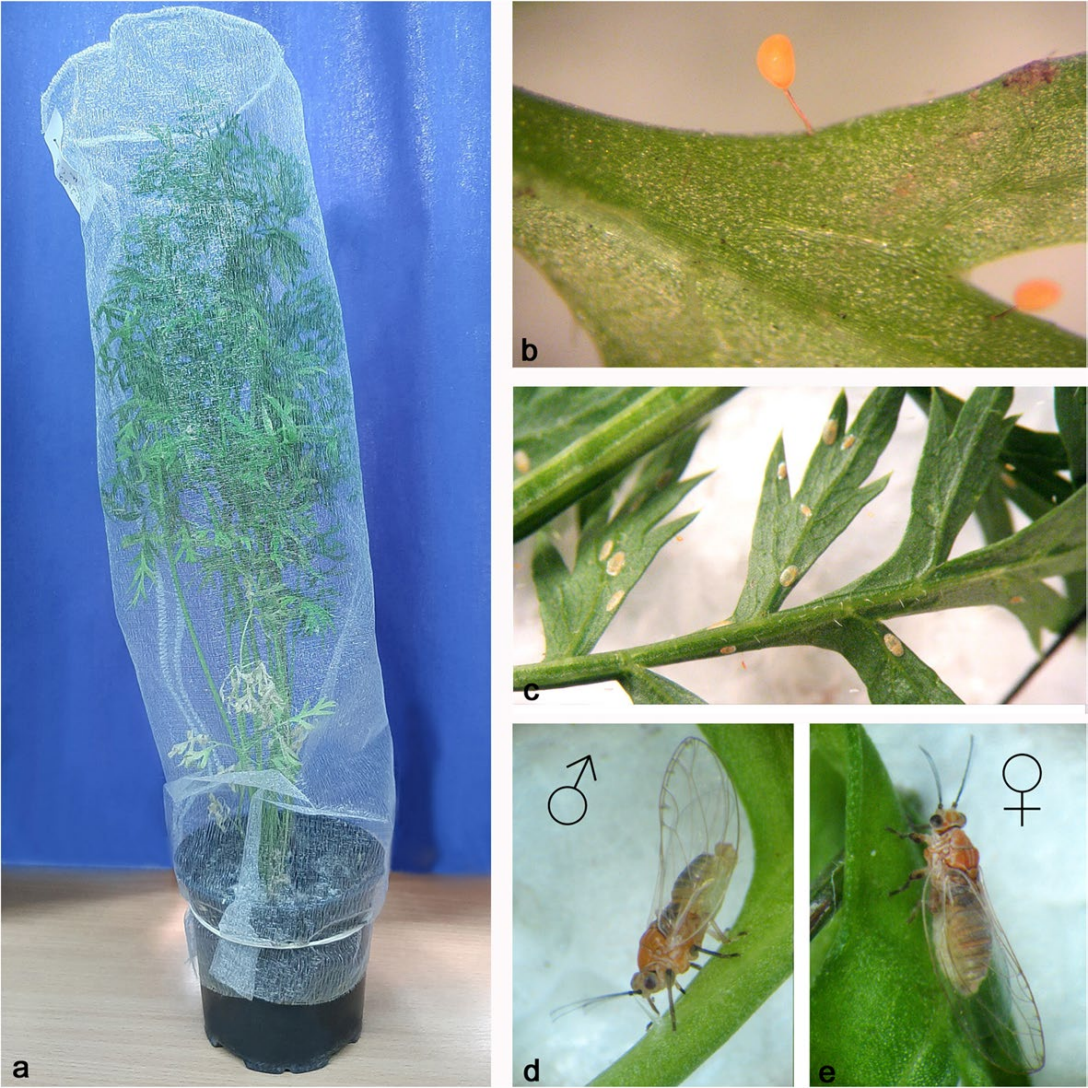
The *B. trigonica* farming overview. **a** Carrot plant infested by *B. trigonica* covered with insect net; **b** Detail of a carrot leaf with *B. trigonica* eggs; **c** Abaxial surface of a carrot leaf with dorsoventrally flattened *B. trigonica* nymphs; **d** Male of *B. trigonica*; **e** Female of *B. trigonica*

*B. trigonica* was collected in April in the locality of Begeč (45^0^14′25 "N 19^0^39′35 "E), Serbia. The locality is an area of intensive carrot cultivation, where agricultural techniques and chemical control of weeds, diseases and pests are used. The collected imago individuals were examined and selected under a binocular microscope with a 40 × magnification, and inoculated into each cultivated carrot plant and cultured as described in Jerinić-Prodanović et al. [19]. *B. trigonica* males (♂) and females (♀) (5 individuals each) were inoculated onto carrot plants covered with insect net and grown under controlled conditions (Fig. 12a). After a few days, numerous asymmetric yellow *B. trigonica* eggs are visible on long stalks embedded in plant tissue (Fig. 12b). After seven days, the first nymphal stages hatch (Fig. 12c), and after about 32 days, the next generation of male (Fig. 12d) and female (Fig. 12e) has emerged. In our experiments, these carrot plants represent the parental control plants (PC), which serve as the source of insects used in further experiments and as positive controls.

### Experiment design

Three separate experiments were conducted to evaluate the physiological and oxidative stress response of carrot to *B. trigonica* infestation.

In the first experiment, male and female *B. trigonica* individuals were inoculated on the carrot plants separately or together for one hour each. Four treatment groups were formed to evaluate the effects of insect genders: control plants without *B. trigonica* infestation as a negative control (NC); two groups of carrot plants inoculated with male (6 imago) or female (6 imago) *B. trigonica*; and carrot plants inoculated with both male and female *B. trigonica* (6 imago, each). One hour after *B. triconica* infestation, leaf tissue samples were collected for further physiological and biochemical analyses. In the second experiment carrot plants (*cv*. Nantes) were infested with *B. trigonica* male and female individuals together and all physiological and biochemical parameters were analyzed after short-term infestation (first six hours). In these experimental groups carrot plants were inoculated with male and female of *B. trigonica* together. Samples of leaf tissue were collected during the first six hours (0, 30, 60, 120, 240 and 360 min) of insects infestation. In these experimental groups, carrot plants were inoculated with 14 male and seven females. The third experiment involved long-term infestation of carrots with male and female *B. trigonica*. In this context, the plant samples were analyzed four and 26 days after insect infestation and four treatment groups are included: NC, PC, and two groups of carrot plants infested with *B. trigonica* for four and 26 days.

In all performed experiments, there were five replicates for each treatment group. The samples collected from different treatment groups were immediately frozen in liquid nitrogen and stored at -80 °C before physiological and biochemical analyses.

### Determination of photosynthetic pigments content

Photosynthetic pigments (chlorophyll a, chlorophyll b, and carotenoids) were extracted from 20 mg of frozen carrot leaf tissue with 96% ethanol (2 ml). Samples were incubated in a water bath (Univeba JP Selecta) at 70 °C for 10 min and cooled in the dark. Pigment content was determined spectrophotometrically by measuring the absorbance of the extracts at 470, 648, and 664 nm (Shimadzu UV-1800, Kyoto, Japan). The total chlorophyll content, their ratio, and the carotenoid content were determined according to the formulas proposed by Lichtenthaler [116].

### Oxidative stress assessment

#### Histochemical localization of superoxide anion radicals (O_2_−) and H_2_O_2_ production

To localize superoxide anion (O_2_^−^) production and hydrogen peroxide (H_2_O_2_) accumulation, the whole leaves of non-infested and *B. trigonica*-infested plants were analyzed. The production of O_2_^−^ was evaluated using the nitroblue tetrazolium (NBT, Sigma-Aldrich, St. Louis, MO, USA) staining method, while H_2_O_2_ accumulation was evaluated using the 3,3'-diaminobenzidine (DAB, Sigma-Aldrich, St. Louis, MO, USA) staining method [117]. The whole leaves of NC, PC and *B. trigonica* infested plants (26 days after infestation) were immersed in NBT solution (0.2% NBT, 50 mM sodium phosphate buffer, pH 7.5) and DAB -HCl (1.25 mg/ml, pH 3.8) for two hours. The samples were incubated at room temperature in the dark. Upon contact with O_2_^_^, the pale yellow NBT polymerized and formed blue formazan precipitates, while DAB formed a deep brown product at sites of endogenous H_2_O_2_ accumulation due to the presence of peroxidases. Some leaf tissues were immersed in buffer or 10 mM ascorbic acid as a staining control. After bleaching the tissue with an acetic acid/glycerol/ethanol solution (1:1:3, v/v/v) at 100 °C, samples were immersed in a glycerol/ethanol solution (1:4, v/v) before analysis using a Leica DMLB 2900 light microscope and the program LAS V4.11.

#### Determination of H_2_O_2_ and MDA content

The H_2_O_2_ content in leaf samples (100 mg) was quantitatively determined according to the spectrophotometric method described by Velikova et al. [118]. To evaluate the H_2_O_2_ content in the samples, the absorbance of the extract was measured at 390 nm. MDA content was determined by the method of Heath and Packer [119], with absorbance measured at 532 and 600 nm. Absorbance of both assays was performed using the ELISA Micro Plate Reader (LKB 5060–006, Winooski, Vermont, USA).

#### Determination of proline content

Free proline content in leaf tissue (250 mg) was determined by the ninhydrin reaction using a modified method described by Carillo and Gibon [120]. All modifications are described in detail by Antonić et al. [121] and Trifunović-Momčilov et al. [122]. The absorbance of the obtained yellow reaction product was measured at 350 and 570 nm. In parallel, the absorbance of samples without ninhydrin is used as a negative control, since numerous other compounds also absorb at 350 and 570 nm. The proline content was determined using the proline standard curve. Absorbance measurements were performed using the ELISA Micro Plate Reader (LKB 5060–006, Winooski, Vermont, USA).

#### Determination of total polyphenol content and DPPH radical scavenging activity

Total polyphenol content (TPC) in carrot leaf samples (200 mg) was determined according to the method proposed by Singleton et al. [123] based on the Folin-Ciocalteu reagent (FC). The method is based on the reaction of the reagent FC with the plant polyphenols, which form a blue colored complex that can be easily quantified spectrophotometrically. The absorbance was measured at 765 nm. Details of the modified FC protocol used in this work were described by Đurić et al. [124].

The radical scavenging ability of leaves of non-infested and *B. trigonica*-infested carrot plants was determined by the DPPH (1,1'-diphenyl-2-picrylhydrazyl) method [125]. DPPH is a stable purple compound that react with antioxidants from plant extracts, and transform into a yellow, nonradical molecule hydrazine that can be quantified spectrophotometrically. The absorbance was measured at 520 nm. Details of the modified DPPH methods used in this work were described by Đurić et al. [124].

#### Antioxidative enzyme activities assays

Total soluble proteins were extracted from leaf tissue (1 g) according to the method described by Milošević et al. [126], while protein content in samples was determined by the Bradford method [127]. Superoxide dismutase activity (SOD) was determined according to Beyer and Fridovich [128], with modifications previously described by Antonić et al. [121]. Peroxidase activity (POX) was determined by monitoring the increase in absorbance at 430 nm for two minutes described in detail by Vuleta et al. [129]. Catalase activity (CAT) was estimated by monitoring the consumption of hydrogen peroxide at 240 nm, with the decrease in absorbance proportional to CAT activity [130]. The method was slightly modified: To 1 ml of a reaction mixture containing 0.05 M K-phosphate buffer (pH 7) and 30% H_2_O_2_, 10 µl of the enzyme extract was added and the decrease in absorbance at 240 nm was measured for two minutes. All enzyme activities were measured using a UV–visible spectrophotometer (Shimadzu UV-160, Kyoto, Japan) and expressed as μmol min^−1^ g^−1^ FW (Ug^−1^ FW).

#### Histological analysis and vitality staining

Fresh leaf pieces from non-infested control plants (NC), the mother plant (PC), and plants after 26 days of *B. trigonica* infestation were used for histological analysis. Leaf tissues were examined by cross-sectioning the plant material by hand with a razor blade prior to staining. Leaf samples were stained with Evan blue staining solution (0.25 g Evan's blue dye dissolved in 0.1 M CaCl_2_ solution, pH 5.6) for several minutes [131]. All samples were analyzed using a light microscope (Leica DMLB 2900 with the program LAS V4.11).

### Statistical analysis

Evaluation of all analyzed parameters was performed on three biological samples per treatment, and results are presented as mean ± standard error. STATISTICA software version 8 was used to evaluate statistical differences between experimental treatments using standard analysis of variance (ANOVA). Mean differences were compared using the least significant difference (LSD) test at a statistical significance of *p* ≤ 0.05. Graphical representation of the results was performed using the Microsoft Office Excel (2010) program.

## Data Availability

The datasets used and/or analyzed during current study are available from corresponding author on reasonable request.
